# Ectopic Pancreatic Tissue in the Gallbladder

**DOI:** 10.31486/toj.23.0120

**Published:** 2024

**Authors:** Manveen Kaur, Aprajita Garg, Rajeev Sharma

**Affiliations:** ^1^ Department of Pathology, Government Medical College and Hospital, Chandigarh, India; ^2^ Department of General Surgery, Government Medical College and Hospital, Chandigarh, India

**Keywords:** *Congenital*, *gallbladder*, *incidental findings*, *pancreas*

## Abstract

**Background:** Ectopic pancreatic tissue, also referred to as heterotopic pancreas, is defined as the presence of pancreatic tissue in an organ outside the pancreas. Ectopic pancreatic tissue, a rare embryologic abnormality, has been reported in the stomach, duodenum, colon, and Meckel diverticulum and is usually discovered incidentally.

**Case Report:** We report a case of ectopic pancreatic tissue in the gallbladder of a 37-year-old female who underwent a cholecystectomy after a clinical diagnosis of chronic cholecystitis. Histopathologic findings were chronic cholecystitis with cholesterolosis and pyloric metaplasia. The gallbladder wall showed ectopic pancreatic tissue composed of acini, ducts, and islets of Langerhans. The histopathologic examination confirmed the diagnosis of ectopic pancreatic tissue.

**Conclusion:** Making a preoperative diagnosis of ectopic pancreatic tissue is clinically and radiologically challenging. Meticulous histopathologic examination is required for the diagnosis of this rare condition.

## INTRODUCTION

Ectopic pancreatic tissue, also referred to as heterotopic pancreas or pancreatic choristoma, is defined as the presence of pancreatic tissue in an anomalous location without any anatomic, vascular, or neural continuity with the main body of the normal pancreas.^1^ Ectopic pancreatic tissue occurs rarely, and despite its congenital origin, is often discovered incidentally, such as during a pathologic examination of a cholecystectomy specimen. The overall incidence ranges from 0.55% to 13.7% discovered in autopsies and <0.2% discovered in laparotomies, with the most common sites being the stomach, duodenum, colon, and Meckel diverticulum.^2-4^ We report a case of incidental detection of ectopic pancreatic tissue in a cholecystectomy specimen removed for symptomatic cholelithiasis.

## CASE REPORT

A 37-year-old female presented to the surgery outpatient department with complaints of pain in the right upper quadrant for the prior 4 months. The pain worsened in intensity after she consumed fatty meals. The patient's medical history was not significant. On general physical examination, she was hemodynamically stable. The right upper quadrant of the abdomen was tender on palpation. Laboratory investigations showed normal biochemical parameters (liver function tests, renal function tests, and lipid profile).

Ultrasound of the abdomen revealed a single 16-mm calculus in the gallbladder, and the patient was scheduled for laparoscopic cholecystectomy with a clinical diagnosis of chronic cholecystitis with cholelithiasis. The intraoperative and postoperative periods were uneventful, and the patient was discharged the day after surgery. The specimen was sent for histopathologic examination.

**Figure 1.**
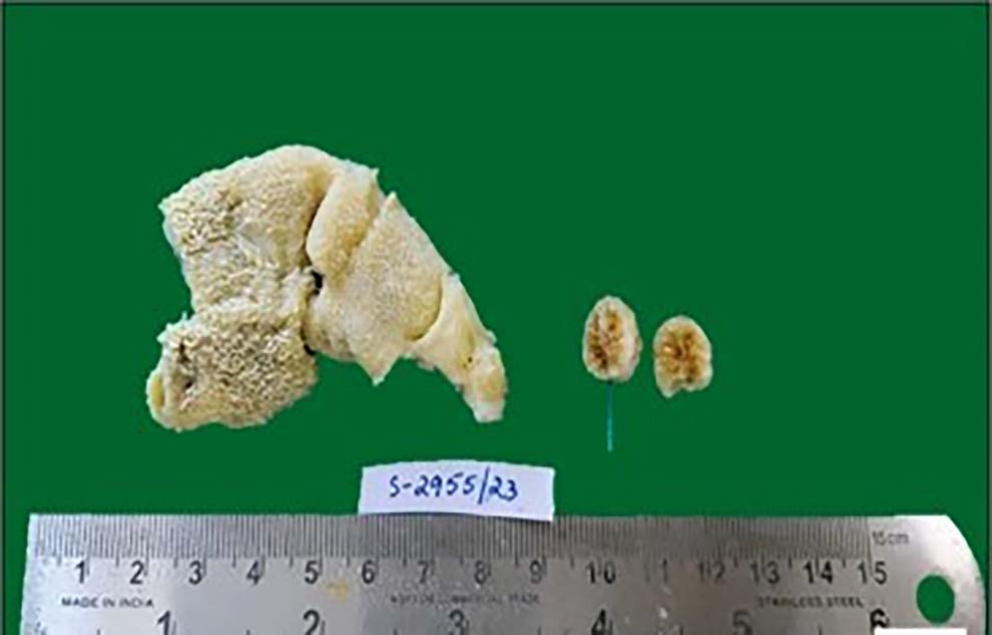
Cholecystectomy specimen measured 9 cm, with a wall thickness of 0.3 to 0.5 cm. Cut section shows bile-stained mucosa with grey-yellow specks and a single cholesterol stone.

Macroscopically, the gallbladder specimen measured 9 cm and had a single cholesterol stone. Gallbladder wall thickness measured 0.3 to 0.5 cm (Figure 1). Mucosa was bile stained with grey-yellow specks.

**Figure 2.**
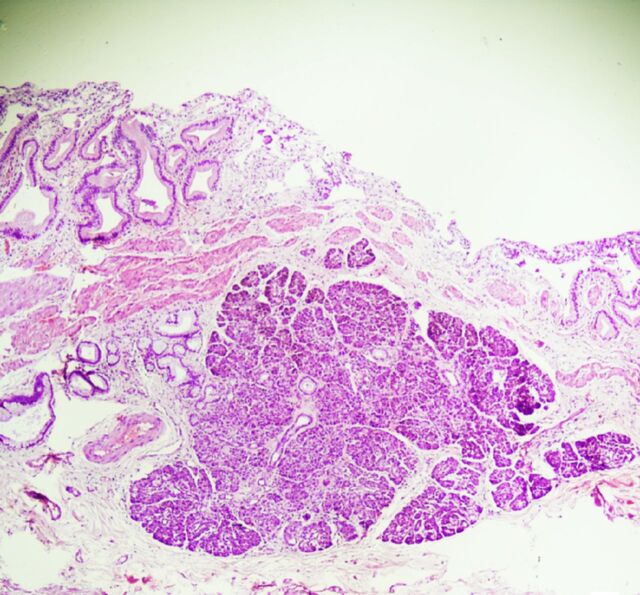
Photomicrograph shows formation of Rokitansky-Aschoff sinuses and evidence of pyloric metaplasia. Lamina propria shows foamy macrophages and chronic inflammatory cell infiltrate (hematoxylin and eosin stain, magnification × 40).

Sections from different parts of the gallbladder (ie, fundus, body, and neck) were submitted for microscopic analysis. Microscopic examination revealed mucosa lined by tall columnar epithelium with chronic inflammation changes in the form of Rokitansky-Aschoff sinuses and evidence of pyloric metaplasia. Lamina propria showed foamy macrophages and chronic inflammatory cell infiltrate corresponding to the grey-yellow specks seen on gross examination (Figure 2). The findings were characteristic of chronic cholecystitis with cholesterolosis and focal pyloric metaplasia.

**Figure 3.**
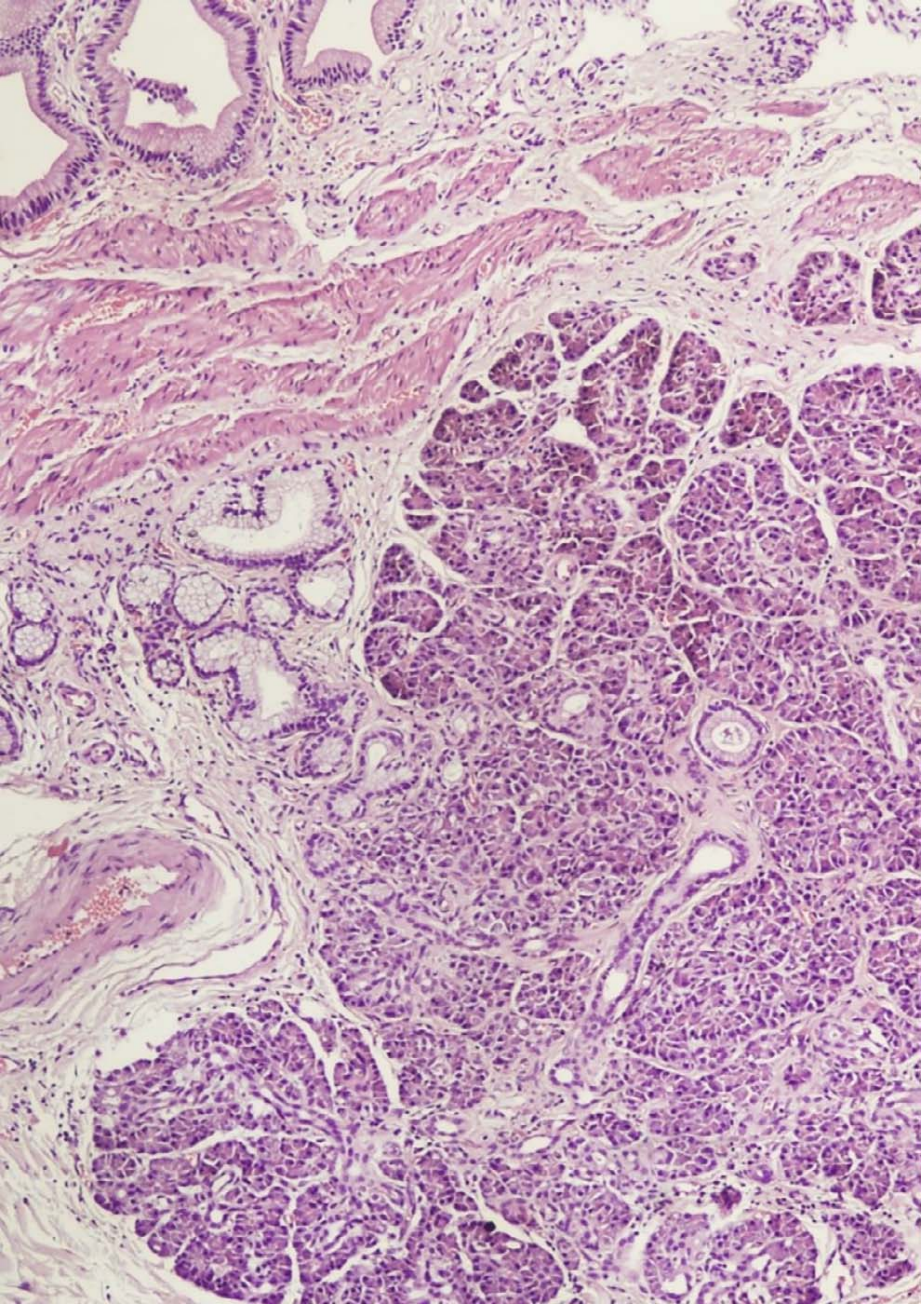
Photomicrograph shows ectopic pancreatic tissue in the wall of the gallbladder (hematoxylin and eosin stain, magnification × 200).

In addition, the wall of the gallbladder showed ectopic pancreatic tissue composed of acini, ducts, and islets of Langerhans (Figure 3). The acini were composed of clusters of cells lined by cuboidal epithelium. The cells showed basally placed nuclei and basophilic granular cytoplasm. No evidence of dysplasia or malignancy was found. Microscopically, the size of the ectopic pancreatic tissue was estimated to be 2 mm. The thickening of the gallbladder wall could be attributed to the chronic inflammation and the ectopic pancreatic tissue. Our case was classified as Type 4 according to the modified von Heinrich classification of ectopic pancreas.^5,6^

At 1-month follow-up, the patient reported relief from her preoperative symptoms and was doing well. Because no microscopic evidence of dysplasia was found in the gallbladder specimen, the patient required no further follow-up.

## DISCUSSION

The first case of ectopic pancreatic tissue in the gallbladder was published in 1916,^7^ and as of 2021, fewer than 40 cases had been reported.^8^ As stated previously, the common sites for ectopic pancreatic tissue are in the gastrointestinal tract, including the stomach, duodenum, colon, and Meckel diverticulum, whereas the less commonly involved sites are the spleen, liver, omentum, lungs, fallopian tubes, and gallbladder.^3,4^

Various theories have been advanced to explain the development of ectopic pancreatic tissue in the gallbladder. The most accepted ones are misplacement theory and metaplastic theory.^9-11^ Misplacement theory suggests that ectopic pancreatic tissue occurs because of the early separation of the pancreas during the rotation of the gastrointestinal tract during the embryologic period.^9-11^ Metaplastic theory suggests that the longitudinal growth of the intestines is responsible for the migration of some cells from pancreatic buds, leading to ectopic pancreatic tissue in the gallbladder.^9-11^ In 2006, Fukuda et al posited that alterations in the Notch signaling pathway lead to differentiation during the development of the endoderm of the foregut.^12^

In the early 1900s, Heine von Heinrich proposed a 3-tier classification for the pathologic findings of ectopic pancreatic tissue.^5^ Gaspar Fuentes et al later modified this system into a 4-tier classification: Type 1, presence of acini, ducts, and islet-like pancreatic gland; Type 2, canalicular variant with pancreatic ducts; Type 3, exocrine pancreas with acinar tissue; and Type 4, endocrine pancreas with cellular islets.^6^ Our patient had endocrine pancreatic tissue with islets and therefore was classified as Type 4.

Ectopic pancreatic tissue is reported to have a female predominance, as surgical gallbladder pathology is found more commonly in females than males.^8^ Most patients with ectopic pancreatic tissue are asymptomatic, but in some cases, patients may present with nonspecific symptoms such as epigastric pain, abdominal distension, melena, or vomiting.^3^ Patients may also present with clinical symptoms of acute pancreatitis or, in rare cases, with peritonitis. Also, ectopic pancreatic tissue may be complicated by inflammation, bleeding, obstruction, or malignant transformation.^3,9^

The preoperative diagnosis of ectopic pancreatic tissue is clinically and radiologically challenging. Imaging modalities such as ultrasound or computed tomography are not helpful in diagnosing ectopic pancreatic tissue, and ectopic pancreatic tissue may resemble cholesterol polyps, cholecystitis, cysts, abscesses, adenomyomas, or even carcinomas. Lesion size may range from a few millimeters to as large as 4 cm.^13,14^ Our patient had a very small focus of ectopic pancreatic tissue that could not be detected on radiologic examination. Even gross examination of the specimen did not reveal any definite subepithelial lesion, although the thickness of the gallbladder wall was mildly increased.

Ectopic pancreatic tissue may undergo metaplasia or even neoplastic transformation that affects the normal pancreas.^14^ However, because of the low rate of malignant transformation, ranging from 0.7% to 1.8%, current recommendations do not support routine biopsy or follow-up screening for incidental ectopic pancreatic tissue.^14^ In 2017, Vitiello et al reported that an effective treatment for symptomatic ectopic pancreatic lesions >3 cm is endoscopic mucosal resection and endoscopic submucosal dissection.^15^ In most cases, surgery is the treatment of choice for the relief of symptoms and to diagnose or exclude malignancy.

## CONCLUSION

Ectopic pancreatic tissue in the gallbladder is a rare finding and is usually detected incidentally. Meticulous histopathologic examination with sections from different parts of the gallbladder is required to diagnose this condition.

## Notes

*This article meets the Accreditation Council for Graduate Medical Education and the American Board of Medical Specialties Maintenance of Certification competencies for Patient Care and Medical Knowledge.*
